# Cerebral Lymphangioma: A Clinical Case History

**DOI:** 10.7759/cureus.86455

**Published:** 2025-06-20

**Authors:** Fadwa Fliyou, Sidi Mamoun Louraoui, Youssef Ouboukhlik, Mounir Rghioui, Abdessamad El Azhari, Faycal Moufid

**Affiliations:** 1 Neurosurgery, Cheikh Khalifa International University Hospital, Mohammed VI University of Sciences and Health (UM6SS), Casablanca, MAR; 2 Neurosurgery, Cheikh Khalifa International University Hospital, Casablanca, MAR; 3 Neurosurgery, Mohammed VI International University Hospital, Mohammed VI University of Sciences and Health (UM6SS), Casablanca, MAR

**Keywords:** brain tumor, cerebral lymphangioma, cystic lymphangioma, intracranial cyst, lymphatic malformation

## Abstract

Cerebral lymphangioma is an extremely rare entity, especially in adults. We report the case of a 38-year-old male who presented with diabetic ketoacidosis (fasting glucose: 4.65 g/L; glycated hemoglobin (HbA1c): 9.5%; urinary ketones and glucose: 4+ (highly positive)), severe holocranial headache, left hemiparesis, and agitation. MRI revealed a 58×46.5 mm right parietal extra-axial lesion with cystic and hemorrhagic components, initially suggestive of a cystic meningioma. Gross total surgical resection was performed following metabolic stabilization. Histopathology confirmed the diagnosis of lymphangioma. Postoperatively, the patient experienced a marked reduction in headache intensity (from 8/10 to 2/10 on the visual analog scale) and improvement in motor strength.

This case illustrates the diagnostic challenge posed by cerebral lymphangiomas, which may closely mimic other cystic extra-axial lesions on imaging, particularly hemorrhagic meningiomas. It also demonstrates that complete surgical resection can result in favorable clinical outcomes. To our knowledge, this is one of the very few documented cases of cerebral lymphangioma in an adult, underscoring the need for increased awareness and further case reports to guide diagnosis and management.

## Introduction

Cystic lymphangioma is a benign malformation of the lymphatic system [1], most often diagnosed in early childhood, with over 90% of cases occurring before the age of two [2,3]. These lesions typically affect the cervicofacial region (75%) and axillary region (15%) and are rarely encountered outside of these areas [4,5].

In contrast, cerebral lymphangioma (CL) is an exceptionally rare entity, with only one similar case reported to date in the literature. Its occurrence in adults, especially in a supratentorial intracranial location, is highly unusual and presents a significant diagnostic challenge, often mimicking other more common intracranial lesions such as hemorrhagic meningiomas or cystic tumors.

This report describes a rare case of adult-onset cerebral lymphangioma, managed successfully by total surgical resection. The case stands out by its atypical location, its radiological presentation, and the absence of lymphangiomatous lesions in classical sites. Through this observation, we aim to contribute to the extremely limited body of knowledge on intracranial lymphangiomas and provide insights into their pathogenesis, clinical presentation, differential diagnosis, and management strategies.

## Case presentation

A 47-year-old man with type 2 diabetes mellitus treated with oral agents presented with severe holocranial headache, dizziness, vomiting, agitation, and a sensation of heaviness on the left side of his body. A neurological examination demonstrated left-sided hemiparesis graded 4/5 on the Medical Research Council scale.

Laboratory tests confirmed diabetic ketoacidosis (DKA): fasting blood glucose, 4.65 g/L (465 mg/dL); glycated hemoglobin (HbA1c), 9.5%; and urine dipstick, 4+ (highly positive) ketones and 4+ (highly positive) glucose. Although arterial blood gas was not obtained, the clinical and biochemical profile indicated moderate to severe metabolic decompensation.

**Figure 1:**
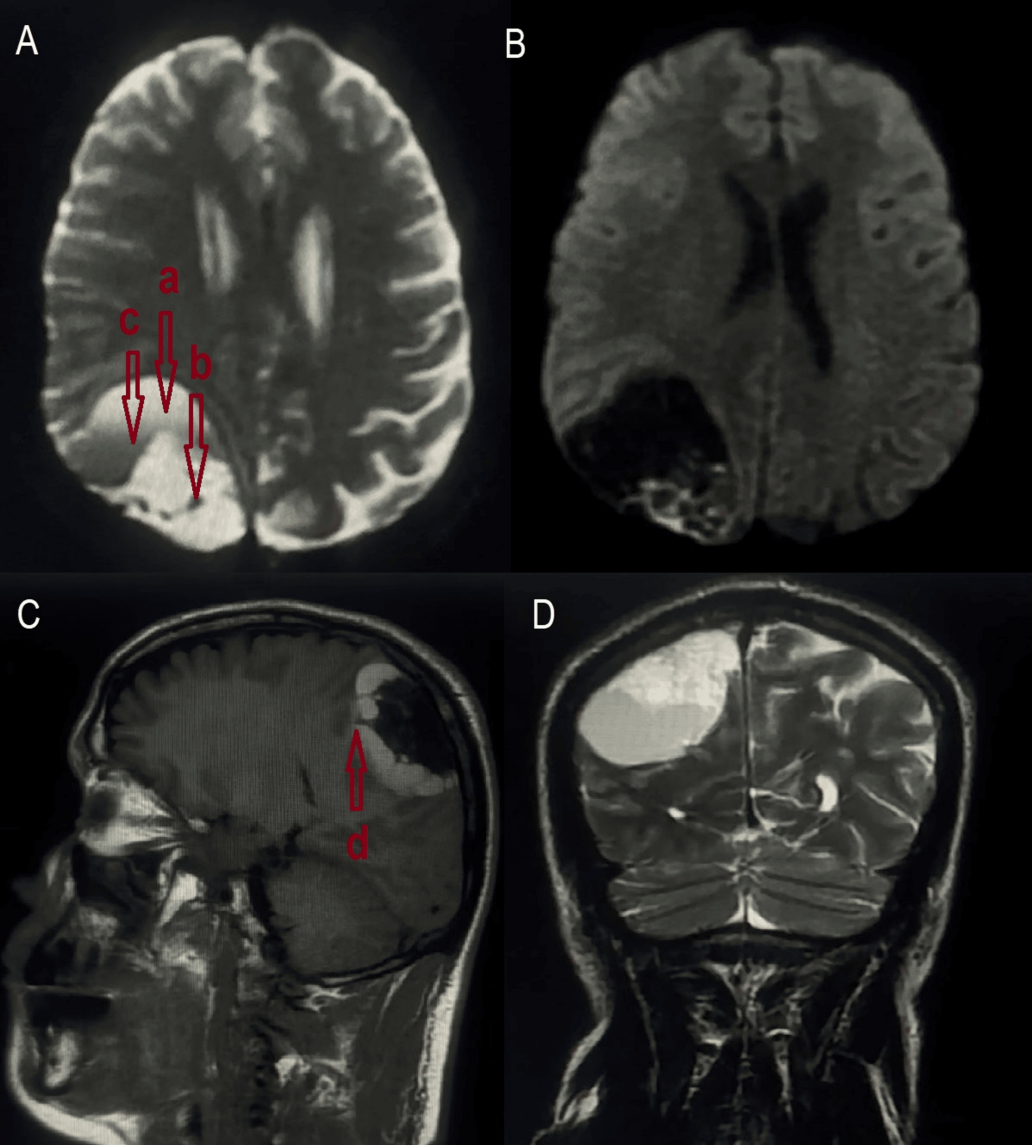
Brain MRI revealed a right parietal lesion with a broad dural attachment. It was predominantly cystic, featuring a posterior solid component exhibiting low signal intensity on T1-weighted images (A, C) and high signal intensity on T2-weighted images (D). The anterior cystic portion displayed hemorrhagic characteristics, with high signal intensities on T1, T2, and FLAIR sequences, alongside a fluid-fluid level indicative of rebleeding (A, D). a: Cystic portion of the lymphangioma; b: Solid portion of the lymphangioma; c: Fluid-fluid level indicating rebleeding; d: Septa partitioning the cystic part of the lymphangioma FLAIR: fluid-attenuated inversion recovery

Brain MRI showed a 58 × 46.5 mm right parietal extra-axial lesion with a broad dural attachment. The mass was predominantly cystic, with a posterior solid component that was hypointense on T1-weighted images, hyperintense on T2, and exhibited diffusion restriction on apparent diffusion coefficient (ADC) maps. The anterior cystic portion displayed hemorrhagic features: high signal on T1, T2, and FLAIR, and a fluid-fluid level suggestive of recent rebleeding. The absence of contrast enhancement led to an initial impression of cystic meningioma (Figure 1).

**Figure 2:**
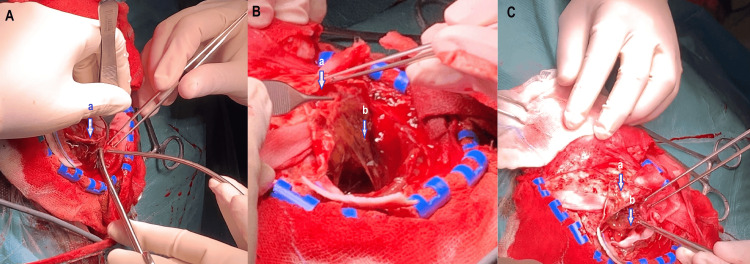
Intraoperative images showing the resection of an intracranial lesion located intradurally and extra-axially (A), with both cystic (B) and solid (C) components a: Dura mater; b: Cystic portion of the lymphangioma; c: Fleshy portion of the lymphangioma

**Figure 3:**
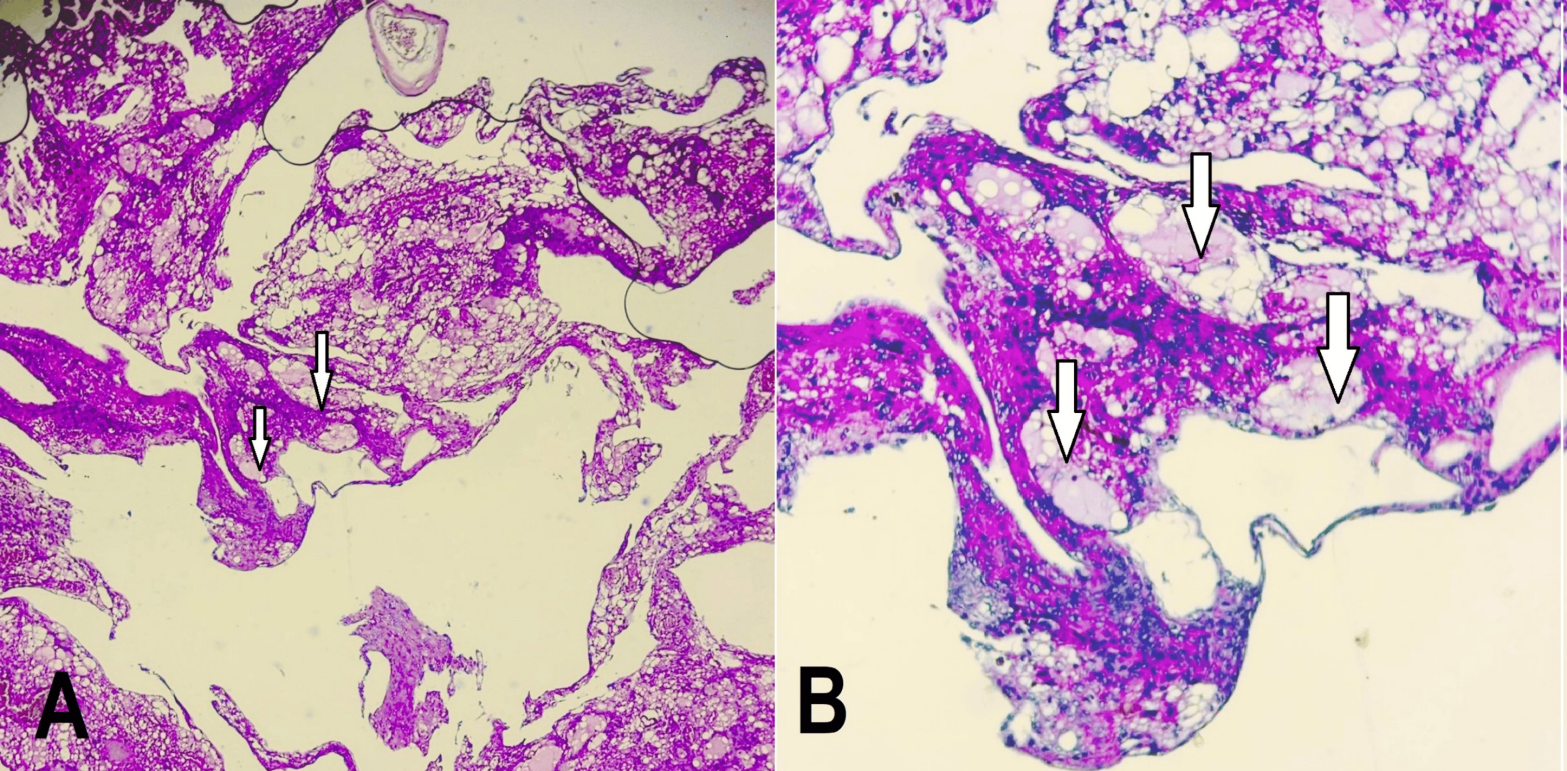
Histopathological images of the tumor showing (A) a vascular proliferation composed of frequently ectatic vessels with an endothelial‐type lining (HE×10) and (B) this vascular proliferation consisting of lymph‐filled vessels with flattened walls (HE×40)

After metabolic stabilization, the patient underwent a right parietal craniotomy. Under neuronavigation guidance, a horseshoe-shaped scalp incision and a tailored craniotomy centered on the lesion were performed. Careful dural opening exposed a well-circumscribed cystic mass lying adjacent to, but not infiltrating, the dura mater and superior sagittal sinus. The cyst contained hemorrhagic fluid and friable, pinkish tissue. Gross total resection was achieved with meticulous dissection, preserving the dura, the sinus, and the adjacent cerebral parenchyma (Figure 2). No abnormal adhesions or vascular involvement were encountered. Hemostasis was obtained without the need for dural reconstruction. The bone flap was replaced and secured. The estimated blood loss was 300 mL, and the operative time was approximately 110 minutes. There were no intra or postoperative complications. Figure 3 shows the histopathological images of the tumor.

**Figure 4:**
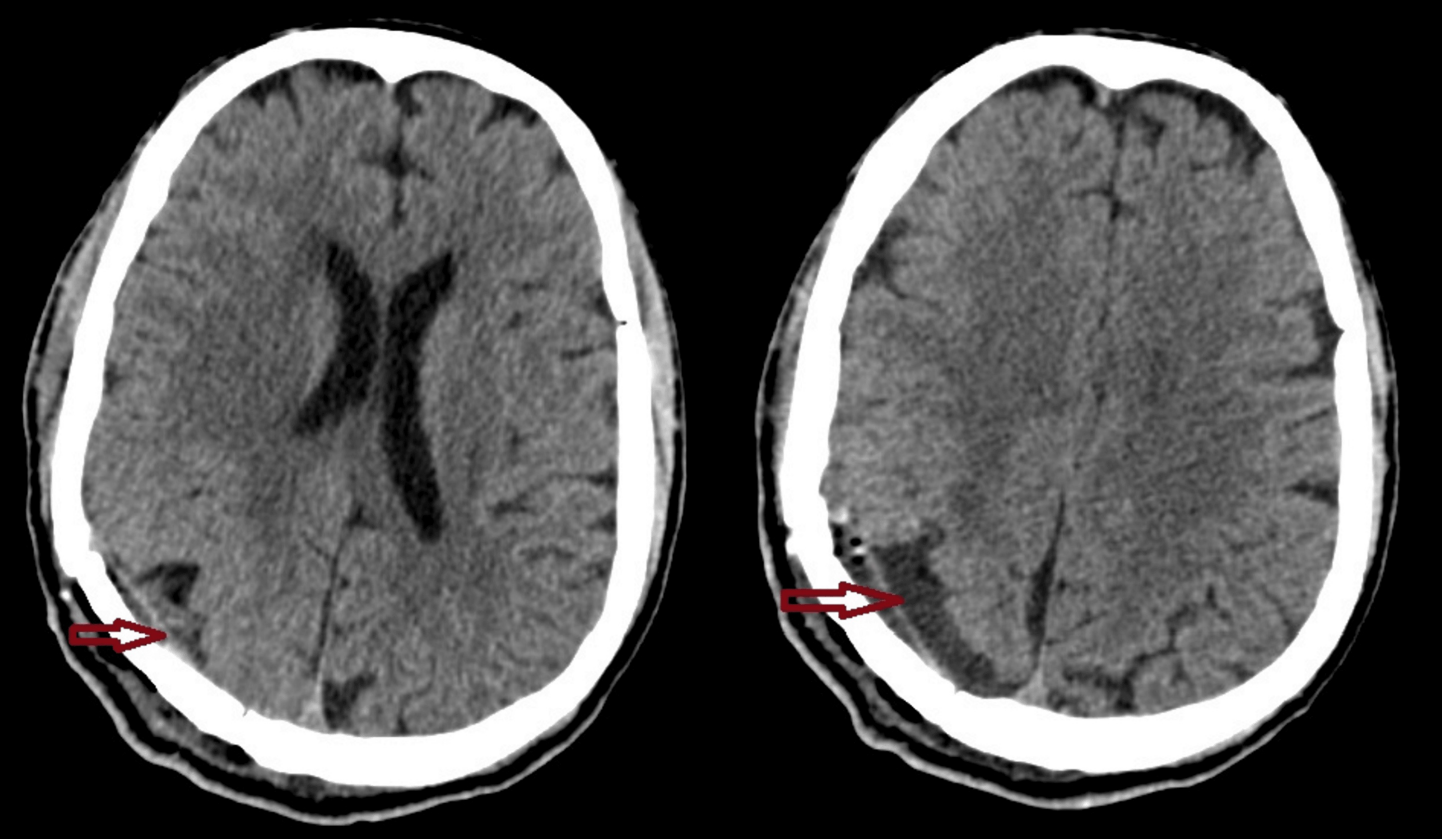
A postoperative CT scan confirmed complete lesion resection

Postoperatively, the patient’s headache score improved from 8/10 to 2/10 on the visual analog scale, and motor strength returned to baseline. CT confirmed complete tumor removal (Figure 4). The patient’s recovery was uneventful, and he was discharged on postoperative day 7.

Postoperatively, the patient’s headache score improved from 8/10 to 2/10 on the visual analog scale, and motor strength returned to baseline. CT confirmed complete tumor removal (Figure 4). The patient’s recovery was uneventful, and he was discharged on postoperative day 7.

## Discussion

Cerebral lymphangioma is an extremely rare entity [2,6], with only one previously reported case by a neurosurgical team in Tunis, describing an adult patient with a parietal lesion. To our knowledge, this case was presented in Tunisia as an oral communication during a scientific meeting at the National Institute of Neurology in Tunis in 2014, but it has not been formally published. This rarity makes our case particularly noteworthy, especially given the adult-onset, atypical radiological features mimicking a cystic meningioma and the hemorrhagic nature of the lesion.

Unlike the more common cervicofacial lymphangiomas, which constitute the majority of lymphatic malformations and are typically diagnosed in early childhood [4,5], intracranial lymphangiomas remain poorly characterized. Their pathogenesis is presumed to mirror that of peripheral lesions, involving congenital malformations of lymphatic channels [6,7]. However, the absence of lymphatic vessels in the normal brain raises questions about their true embryological origin, with some authors suggesting heterotopic lymphatic rests or lymphatic proliferation following inflammation or trauma as possible explanations [8-10].

The clinical presentation in cerebral cases varies with lesion size and location. In our patient, the tumor caused signs of intracranial hypertension, focal neurological deficits, and a diabetic ketoacidosis crisis, likely precipitated by systemic stress. Radiologically, the lesion mimicked a cystic meningioma due to its extra-axial location, dural attachment, and mixed cystic and solid components. However, the presence of fluid-fluid levels and hemorrhagic signals raised suspicion for an alternative diagnosis [11,12]. The absence of contrast enhancement further complicated preoperative identification [13,14]. Compared to the Tunisian case, which involved a similarly located lesion, our case adds further evidence of a potential predilection for the parietal region. The Tunisian case presented a more complex lesion with both cystic and solid components, hemorrhagic changes, calcifications, and involvement of the ventricular system. In contrast, our case featured a well-defined, extra-axial cystic lesion with fluid-fluid levels but no ventricular invasion or edema. The differences in imaging characteristics may reflect variations in lymphatic malformation subtype, chronicity, or possible secondary changes such as hemorrhage or infection. The Tunisian case emphasizes the potential for lymphangiomas to mimic more aggressive or infiltrative processes due to their heterogeneous appearance. Both cases underscore the importance of including lymphangioma in the differential diagnosis of atypical intracranial cystic lesions, especially in the presence of calcifications or hemorrhage. Histopathological confirmation remains indispensable for definitive diagnosis. Both patients underwent surgical resection with good outcomes, suggesting that complete excision is both feasible and effective in carefully selected cases.

Histological confirmation remains essential for diagnosis, typically revealing large lymphatic channels lined by flat endothelium and filled with serous or hemorrhagic content. Immunohistochemical markers such as D2-40 and Prox1, though not available in our center, can support the lymphatic lineage [15].

Treatment of cerebral lymphangiomas is not standardized due to their rarity. Surgical resection remains the mainstay in symptomatic patients, particularly when mass effect or hemorrhage is present. In our case, gross total resection was achieved without complications. Sclerotherapy, widely used for cervicofacial lymphangiomas, was not considered due to the accessibility of achieving maximal surgical resection in both cases, the hemorrhagic nature of the lesion, and its proximity to major venous structures [16]. Radiosurgery has not yet been evaluated in this context, and its role remains hypothetical.

Given the paucity of reported intracranial lymphangiomas, further documentation is essential to better define their clinical behavior and guide treatment. Long-term surveillance is warranted to monitor for recurrence or delayed complications.

## Conclusions

Cerebral lymphangioma is an exceptionally rare and often unanticipated diagnosis in adults presenting with intracranial cystic lesions. Its clinical and radiological features may closely mimic more common entities, such as cystic meningiomas, highlighting the importance of including it in the differential diagnosis of atypical extra-axial masses. While MRI provides valuable preoperative clues, definitive diagnosis relies on histopathological examination. Surgical resection remains the preferred treatment in symptomatic cases and can lead to excellent functional recovery.

Given the rarity of intracranial lymphangiomas, there is currently no standardized management protocol. To address this gap, the establishment of multicenter case registries and collaborative networks is essential. Such efforts would enable the aggregation of clinical data, facilitate comparative analysis of treatment outcomes, and support the development of evidence-based guidelines for diagnosis and management. In the meantime, continued reporting of individual cases remains vital to improve clinical recognition and optimize patient care.

## References

[1] Ammari W, Berriche O (2015). Orbital cystic hygroma invading the eyeball: report of a case [Article in French]. Pan Afr Med J.

[2] Nadour K, Moujahid M (2016). Cervicothoracic cystic lymphangioma: about a case [Article in French]. Pan Afr Med J.

[3] N’dri K, Adjenou V, Konan A, Gbazi GC, Mensah GD, Aguehounde C, Abby BC (1996). Cervical cystic lymphangioma: contribution of ultrasound and computed tomography to a case [Article in French]. Med Afr Noire.

[4] Bailey CM (1990). Cystic hygroma. Lancet.

[5] Puig S, Casati B, Staudenherz A, Paya K (2005). Vascular low-flow malformations in children: current concepts for classification, diagnosis and therapy. Eur J Radiol.

[6] Colangeli W, Facchini V, Kapitonov A, Zappalà M, Bozza F, Becelli R (2020). Cystic lymphangioma in adult: a case report and a review of the literature. J Surg Case Rep.

[7] Lerat J, Bisdorff-Bresson A, Borsic M (2019). SFORL recommendations (short version) on cervical lymphatic malformations in adults and children: diagnosis [Article in French]. Eur Ann Otorhinolaryngol Head Neck Dis.

[8] Zhou Q, Zheng JW, Mai HM (2011). Treatment guidelines of lymphatic malformations of the head and neck. Oral Oncol.

[9] Elshaar K, AbuAleid L (2019). Adult-onset giant cervical cystic hygroma with pressure manifestations on aerodigestive tract, managed surgically: reporting of a rare case. Ann R Coll Surg Engl.

[10] Dokania V, Rajguru A, Kaur H (2017). Sudden onset, rapidly expansile, cervical cystic hygroma in an adult: a rare case with unusual presentation and extensive review of the literature. Case Rep Otolaryngol.

[11] Smith RJ, Burke DK, Sato Y, Poust RI, Kimura K, Bauman NM (1996). OK-432 therapy for lymphangiomas. Arch Otolaryngol Head Neck Surg.

[12] Moser T, Chapot R, Jahn C, Salvador D, Baldi S, Beaujeux R (2005). Imaging of soft tissue vascular anomalies: diagnosis and treatment [Article in French]. Feuillets Radiol.

[13] Güneyli S, Ceylan N, Bayraktaroğlu S, Acar T, Savaş R (2014). Imaging findings of vascular lesions in the head and neck. Diagn Interv Radiol.

[14] Higgins LJ, Koshy J, Mitchell SE, Weiss CR, Carson KA, Huisman TA, Tekes A (2016). Time-resolved contrast-enhanced MRA (TWIST) with gadofosveset trisodium in the classification of soft-tissue vascular anomalies in the head and neck in children following updated 2014 ISSVA classification: first report on systematic evaluation of MRI and TWIST in a cohort of 47 children. Clin Radiol.

[15] Handa R, Kale R, Upadhyay KK (2004). Isolated médiastinal lymphangioma herniating through the intercostal space. Asian J Surg.

[16] Bisdorff A, Mulliken JB, Carrico J, Robertson RL, Burrows PE (2007). Intracranial vascular anomalies in patients with periorbital lymphatic and lymphaticovenous malformations. AJNR Am J Neuroradiol.

